# Predictive Factors of Secondary Normocalcemic Hyperparathyroidism after Roux-en-Y Gastric Bypass

**DOI:** 10.1155/2018/5010287

**Published:** 2018-03-06

**Authors:** Claudio Casella, Sarah Molfino, Francesco Mittempergher, Carlo Cappelli, Nazario Portolani

**Affiliations:** ^1^Department of Molecular and Translational Medicine, Surgical Clinic, University of Brescia, Brescia, Italy; ^2^Department of Clinical and Experimental Sciences, Surgical Clinic, University of Brescia, Brescia, Italy; ^3^Department of Clinical and Experimental Sciences, Endocrine and Metabolic Unit, Clinica Medica, University of Brescia, Brescia, Italy

## Abstract

**Objective:**

Aim of this study is to evaluate determinants of secondary normocalcemic hyperparathyroidism (SNHPT) persistence in patients who have undergone Roux-en-Y gastric bypass on vertical-banded gastroplasty.

**Methods:**

226 consecutive patients submitted to bariatric surgery were prospectively enrolled and divided in two groups on the basis of preoperative presence of SNHPT. For each patient, we evaluated anthropometric and laboratory parameters. Calcium metabolism (calcemia, PTH, and 25-hydroxy vitamin D serum levels) was studied before surgery and at 6-month intervals (6, 12, and 18 months) as surgical follow-up.

**Results:**

Based on presurgical SNHPT presence or absence, we defined *group 1—*201 patients and *group 2—*25 patients, respectively. Among the *group 1*, 153 (76%) recovered from this endocrinopathy within 6 months after surgery (*group 3*), while the remaining 48 patients (24%) had persistent SNHPT (*group 4*). Comparing the anthropometric and laboratory data of *group 3* with *group 2*, the only statistically significant factor was the elapsed time since a prior effective medically controlled diet that led to a steady and substantial weight loss. We found also a statistically significant difference (*p* < 0.05) between *group 3* and *group 4* in term of % of weight loss and PTH levels.

**Conclusions:**

Patients suitable for bariatric surgery must have history of at least one efficient medically controlled diet, not dating back more than 5 years before surgery. This elapsed time represent the cut-off time within which it is possible to recover from SNHPT in the first semester after Roux-en-Y gastric bypass on vertical-banded gastroplasty. The treatment of vitamin D insufficiency and the evaluation of SNHPT before bariatric surgery should be recommended. The clinical significance of preoperative SNHPT and in particular SNHPT after bariatric surgery remains undefined and further studies are required.

## 1. Introduction

Patients with obesity have a higher risk than population developing nutritional deficiencies, regardless of whether they undergo bariatric surgery [1–7]. Patients with a history of malabsorptive and metabolic procedures show dysregulation of calcium, parathyroid hormone (PTH), and the vitamin D axis [8–20].

One of the common nutritional deficiencies among these groups of patients is secondary normocalcemic hyperparathyroidism (SNHPT) [1, 2, 4–7, 20, 21], characterized by low serum 25-hydroxy vitamin D levels, high PTH levels, and normocalcemia [22].

The prevalence of SNHPT caused by pathological obesity ranges from 25% to 75% [1, 2, 8, 23, 24]. Its etiology is related to patient phototype and ethnicity [9, 22], lack of solar exposure [2, 25], overclothing [2, 25], lack of physical activity [26], low vitamin D and microelement intake (particularly magnesium) [8], high vitamin D storage in adipose tissue and muscle [27, 28], steatohepatitis [26], and tabagism [28].

The prevalence of SNHPT after bariatric surgery ranges from 17% to 70% [8, 10–19, 29]. Postbariatric surgery SNHPT is a multifactorial condition associated with Afro-American ethnicity [11], female sex [11, 12], latitude of residence [4, 18, 29], season [18], low intake of food rich in calcium, changing food habits after surgery [8, 13], lipid and liposoluble vitamin malabsorption induced by malabsorptive and metabolic procedures [29], type of gastric restriction [16], lack of magnesium intake [15, 16], protein malabsorption [16], and increased bone turn over [16, 17].

The symptomatology of SNHPT is mostly silent. Even when reported, symptoms are nonspecific: general weakness, asthenia, and myalgia [30]. Therefore, the diagnosis must be made using laboratory values: low or insufficient serum 25-hydroxy vitamin D, high PTH levels, and normocalcemia [4, 22, 24, 31, 32].

Because almost 70% of gastric bypass patients experience a reversible and transient SNHPT after surgery, some authors consider this condition as a physiological adaptation to surgery-induced weight loss [4, 11, 18].

The aim of this study is to evaluate the determinants of postbariatric SNHPT persistence in patients who undergo Roux-en-Y gastric bypass on vertical-banded gastroplasty [33].

## 2. Methods

Between January 2011 and June 2012, a total of 302 consecutive morbidly obese patients underwent Roux-en-Y gastric bypass on vertical-banded gastroplasty at the Surgical Department Spedali Civili of Brescia, Italy.

**Figure 1:**
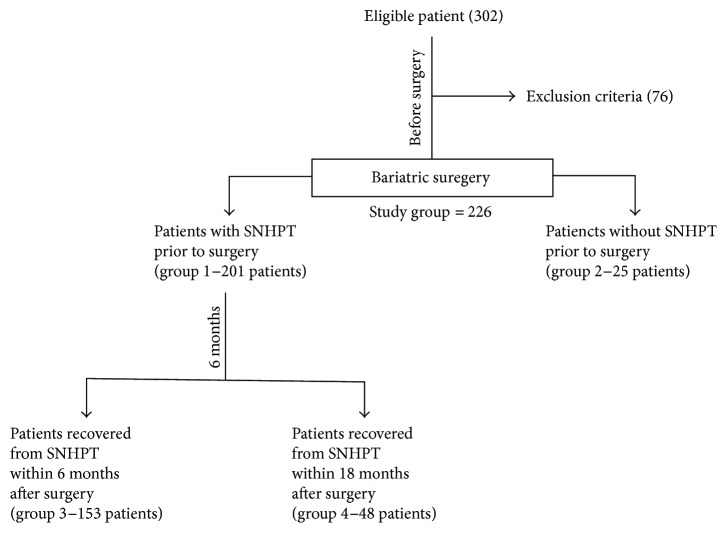
Study design.

A total of 226 (75%) were retrospectively enrolled. Study groups are identified in Figure 1.

The full inclusion and exclusion criteria are listed in Table 1.

**Figure 2:**
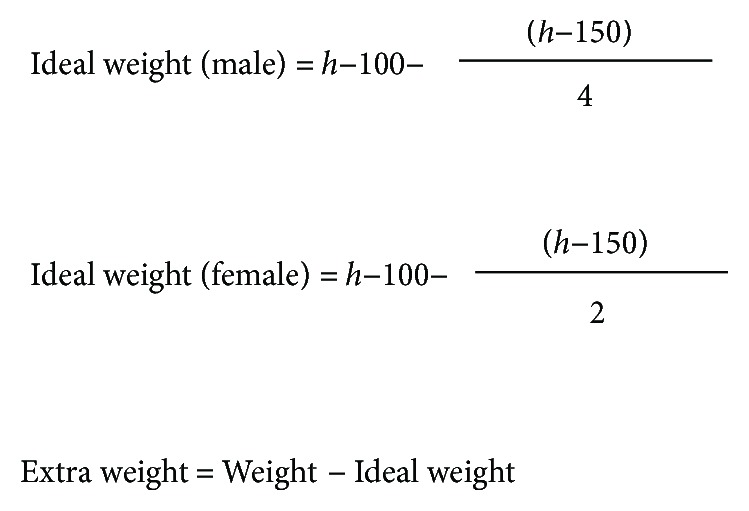
Extra weight defined according to Lorentz formula.

We evaluated anthropometric and laboratory parameters such as age, sex, body mass index (BMI), 25-hydroxy vitamin D3 levels, PTH levels, and serum calcium levels. We also determined the elapsed time since a prior medically controlled diet led to a loss of at least 10% of the initial overweight (calculated according to the Lorentz formula, explained in Figure 2), with subsequent weight maintenance for at least 6 months [34].

A Roux-en-Y gastric bypass on vertical-banded gastroplasty [33] was performed in all patients, fashioning a 50 mL capacity gastric pouch and a common limb 150–200 cm long, with the purpose of keeping the biliopancreatic limb-to-common limb ratio at 1 : 2. Consequently, the biliopancreatic limb had a median length of 23% of the total small intestine lenght.

The particular characteristics of this type of functional gastric bypass (widely performed in Italy but not worldwide) are explained in the article by Cariani et al. [33].

Every patient underwent bariatric surgery according to published Italian guidelines for eligibility [35, 36].

No patients (0%) received oral calcium or vitamin D supplementation before surgery.

All patients (100%) were discharged with the recommendation for oral multivitamin and trace-element supplementation: 1200–2000 mg/die of calcium citrate and at least 3000 IU/day of vitamin D3.

Only patients who have met the study protocol requirements (e.g., oral supplementation if needed) were included.

Serum calcium levels, PTH levels, and 25-hydroxy vitamin D levels were studied before surgery and at 6-month intervals (6, 12, and 18 months). The normal PTH serum concentration was defined from 11 to 67 pg/mL, normal 25-hydroxy vitamin D concentrations from 30 to 60 ng/mL, normal calcium levels from 8.5 to 9.5 mg/dL, and vitamin D deficiency was defined as a serum concentration lower than 20 ng/mL.

Serum calcium levels were assessed by the CA method, in vitro, on the Dimension Vista® System (Siemens). Serum iPTH levels were assessed using the IMMULITE® 2000 System (Siemens). Serum 25-hydroxy vitamin D levels were assessed using the UV/VIS-FAST spectrophotometer (Eureka Lab Division).

Qualitative variables were expressed using the number of patients and percentages; quantitative variables were expressed by using their mean values and standard deviation (SD).

Differences were assessed using analysis of variance (ANOVA) or the Mann–Whitney *U* test for continuous variables and chi-square (𝜒^2^) analysis for categorical variables.

*p* values less than 0.05 were considered statistically significant.

## 3. Results

There were 156 female (69%) and 70 (31%) male patients. Anthropometric and laboratory variables of all patients are listed in Table 2.

Each patient had a history of at least 1 medically controlled diet which failed to result in a steady and substantial weight loss and BMI normalization.

Before surgery, 25 patients (11%) had normal serum PTH levels, 9 patients (4%) had insufficient 25-hydroxy vitamin D levels (20–30 ng/mL), 84 (37%) had vitamin D deficiency (<20 ng/mL), and 133 (59%) had normal vitamin D levels (30–60 ng/mL). All the enrolled patients were normocalcemic. We used this pre-surgical data, to identify 2 subgroups of patients: those with presurgical SNHPT (*group 1—*201 patients) and patients without it (*group 2—*25 patients). (Table 3 and Figure 1).

Table 3 shows that *group 1* and *group 2* were statistically different just because of the level of PTH and the elapsed time since a prior medically controlled diet had led to a loss of at least 10% of the initial overweight, with a weight maintenance for at least 6 months afterward.

Six months after surgery, all *group 2* patients (11%) had maintained calcium-metabolism homeostasis, the oral calcium and vitamin D supplements were suspended. This group of patients never developed SNHPT throughout the duration of study follow-up.

Among the *group 1*, 153 (76%) recovered from this endocrinopathy within 6 months after surgery (*group 3*), while the remaining 48 patients (24%) had persistent SNHPT (*Group 4*), as listed in Table 4.

Comparing the anthropometric and laboratory data of *group 3* with *group 2*, the only statistically significant factor was the elapsed time since a prior effective medically controlled diet that led to a steady and substantial weight loss (Table 4).

Patients who failed to follow an effective medically controlled diet within 5 years before surgery (*group 4*) were found to have higher PTH serum levels than those who followed an effective diet; the SNHPT also lingered for at least 6 months after surgery. By 18 months after surgery, all patients (100%) were able to achieve homeostasis of calcium-metabolism.

Oral supplementation of calcium and vitamin D was suspended 12 months after surgery for the patients who had recovered from SNHPT within the first semester of follow-up (*group 3*). Group 4, however, required an increase in oral supplementation at the 12-month follow-up visit, to 6000 IU/day of cholecalciferol and at least 1800 mg/day of calcium citrate. This supplementation was suspended at the 18-month follow-up visit after confirmation due to laboratory resolution of SNHPT (Table 4).

**Figure 3:**
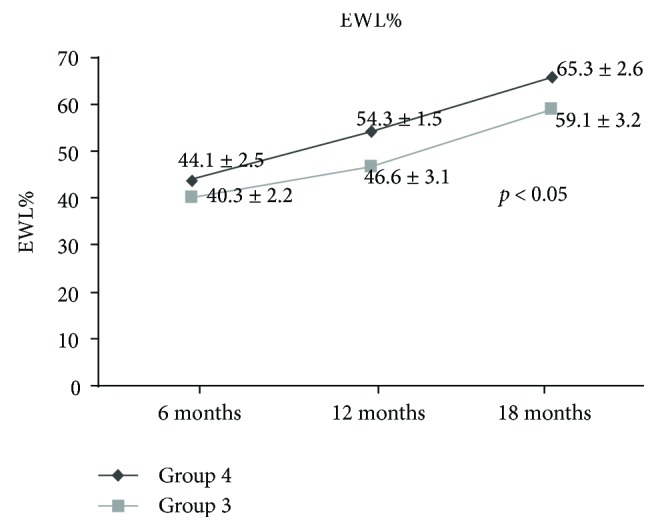
Extra weight loss percentage (EWL%) in the group 3 patients (*n* = 153) recovered from SNHPT within 6 months after surgery and group 4 patients (*n* = 48) within 18 months.

**Figure 4:**
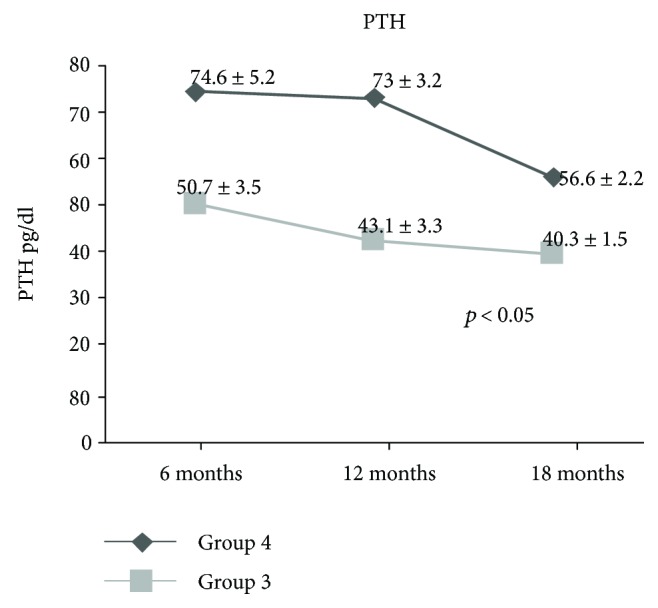
Serum parathyroid hormone levels in group 3 patients (*n* = 153) recovered from SNHPT within 6 months after surgery and group 4 patients (*n* = 48) within 18 months.

Figures 3 and 4 demonstrate a statistically significant difference (*p* < 0.05) between *group 3* and *group 4* in term of % of weight loss and PTH levels, respectively.

## 4. Discussion

The prevalence of pathological obesity-related SNHPT ranges from 25% to 75% [1, 2, 8, 23, 24].

Vitamin D deficiency occurs in 96% of obese patients [1, 2], and that deficit is severe (serum level < 20 ng/mL) in 25.4% [1]. The etiology of SNHPT is multifactorial and relies on patients phototype and ethnicity [9, 22], lack of solar exposure [2, 25], overclothing [2, 25], lack of physical activity [26], low vitamin D and microelement intake (magnesium in particular) [8], high vitamin D storage in adipose tissue and muscle [27, 28], steatohepatitis [26], and tabagism [28].

The particular form of secondary hyperparathyroidism known as SNHPT [1, 2, 4–21] is characterized by low serum 25-hydroxy vitamin D levels, high PTH levels, and normocalcemia [22]. Patients with SNHPT usually have no symptoms; when symptoms are reported, they are often nonspecific, like general weakness, asthenia, and myalgia [30]. Therefore, the diagnosis must be made using laboratory values: low or insufficient serum 25-hydroxy vitamin D levels, high PTH levels, and normocalcemia [4, 22, 24, 31, 32].

An increase of 10 percent body fat is associated with about a 0.4 pmol/L higher serum PTH level after adjustment for age, sex, and calcium intake [21]. Furthermore, every BMI increase of 1 kg/m^2^ leads to a decrease of 1.3 nmol/L of 25-hydroxy vitamin D levels [21]. There is no agreement in literature on the existence of an inverse correlation between PTH and 25-hydroxy vitamin D levels in obese patients [2, 37].

Bariatric surgery results in significant weight reduction and improvement in comorbid obesity-related and general health conditions. Nevertheless, these procedures may concurrently lead to nutritional deficiencies of varying degrees, especially dysregulation of calcium, PTH, and vitamin D axis [8–19, 26, 29]. The prevalence of SNHPT after bariatric surgery ranges from 17% to 70% [8, 10–19, 29].

Premenopausal women (aged > 45 years) have a relative risk of 1.8 compared with younger women of developing SNHPT [11, 12]. The lower postsurgical intake of calcium rich nourishment, due to increase of lactose intolerance and changing in food preferences, contributes to the decreasing serum 25-hydroxy vitamin D levels [8, 13]. Surgically induced lipid and liposoluble vitamin malabsoption, along with gastric restriction [16], reduces their exposure to bile, boosting nutritional deficiency [24]. Magnesium deficiency, typical in bariatric patients, might reduce calcium absorption, which could then lead to SNHPT [15, 16]. Protein, microelements (chromium, copper, selenium, and zinc), essential minerals (iodine and iron), and soluble vitamins (thiamine, cobalamin, riboflavin, and vitamin C) malabsorption may contribute to SNHPT, as may adiponectin, adipokines, and estradiol [16].

Because almost 70% of gastric bypass patients show a reversible and transient SNHPT, during postsurgical follow-up [4, 22, 31, 32], some authors consider this condition to be a physiological adaptation to surgery-induced weight loss [4, 11, 18]. Other authors suggest that bariatric surgery is not the main cause of SNHPT, but rather that this particular form of hyperparathyroidism is indeed a consequence of pathological obesity itself [1, 2, 4, 5, 21, 38].

Laboratory analyses are fundamental to reaching an accurate diagnosis, since this clinical condition is mainly nonspecific and symptomatically silent [30].

There is no agreement onto the efficacy of postsurgical calcium citrate supplementation to reduce the incidence of metabolic disorders [10, 11, 38–41]. Moreover, because SNHPT is most often present prior to bariatric surgery, current guidelines suggest stabilizing this dysmetabolism before surgey [41]. The American Association of Clinical Endocrinologists, the Obesity Society, and the American Society of Metabolic and Bariatric Surgery (AACE/TOS/ASMBS) guidelines suggest oral administration 1500–2000 mg per day of calcium citrate and at least 3000 IU (75 *μ*g) per day of vitamin D2 or D3 after surgery, with the chance to boost the latter administration until reaching serum 25-hydroxy vitamin D levels of >30 ng/mL (75 mmol/L). In cases of severe vitamin D malabsorption, oral doses of vitamin D2 or D3 may need to be as high as 50,000 units 1 to 3 times weekly to daily, and more recalcitrant cases may require concurrent oral calcitriol.

In our study, we established the importance of the presurgical diet in the resolution of SNHPT, especially when a medically controlled diet, dating back no more than 5 years before surgery, led to a loss of at least 10% of the initial overweight with weight maintenance for at least 6 months afterward.

Forty-eight (24%) patients with presurgical SNHPT recovered from this condition within 18 months after surgery. These patients had no effective medically controlled diet in the 5 years before surgery (Table 4) but rather had long-standing morbid obesity. These patients' BMI values had borderline statistically significant elevation compared with patients not affected by SNHPT. After surgery, the patients with presurgical SNHPT had a statistically significant (*p* < 0.05) greater weight loss either than patients who never had SNHPT or patients who recovered within the first semester after bariatric surgery; this difference persisted throughout study follow-up (Figure 3).

Even though the group of patients with presurgical SNHPT recovered from this disorder within 18 months after surgery, their serum PTH levels were statistically significantly (*p* < 0.05) higher than those of other patients throughout the duration of study follow-up. (Figure 4).

## 5. Conclusion

SNHPT is a clinical condition related to morbid obesity [1–6, 9, 21, 25–28] and needs to be stabilized prior to surgery [41].

Patients with obesity suitable for bariatric surgery must have history of at least 1 medically controlled diet, not dating back more than 5 years before surgery, that led to a loss of at least 10% of the initial overweight, with a subsequent weight maintenance for at least 6 months. Patients who had an effective diet within this timeframe may reasonably anticipate a recovery from SNHPT in the first semester after Roux-en-Y gastric bypass on vertical-banded gastroplasty [33].

Patients without this history need a presurgical medically controlled diet and assessment of calcium metabolism. The aim of this diet is not recovery from obesity but the advancement of better postsurgical clinical outcomes and SNHPT resolution. Furthermore, patients must be taught to follow a healthy and balanced lifestyle in order to avoid sudden weight loss that might lead to endocrine disorders. Just as their obesity took time to develop, so should it take time to be healed.

## 6. Final Comments

We evaluated the alterations in the parathyroid axis through its effect on calcium absorption following Roux-en-Y gastric bypass on vertical-banded gastroplasty (RYGBP-on-VBG). Up to date, our findings have not been reported in literature yet. Previous studies investigated the prevalence of SNHPT in obese patients after several bariatric operations but not after RYGBP-on-VBG. Different types of bariatric surgery have different effects on body weight loss and different incidence of SNHPT. How bariatric operations induce SNHPT remains unknown, probably determined by nonsurgical and surgical factors:

- Preoperative status of micronutrient deficit (calcium and vitamin D)
- Decreased postoperative gastric acid secretion leading to impaired intestinal calcium absorption
- Fat malabsorption may affect the absorption of fat soluble vitamins, as vitamin D
- Limited intake of daily products due to intolerance after surgery

In our study we recorded greater postoperative weight loss in patients with preoperative SNHPT.

This finding could be accidental and erroneous, maybe due to the limitations of the study:

- Retrospective study with a relative small sample size
- Lack of food intake analysis among two groups of patients (with presurgical SNHPT and without presurgical SNHPT, group 1 and group 2, resp.)
- Poorness of variable analysis (e.g., the lack of correction for confounding factors due to the unavailability of data as seasonality, physical activity, sun exposure, sunscreen, and phototype)

Other limitations of our study:

- Adherence to vitamin or mineral supplements was indirectly estimated based on patients information
- Unavailability of data such as albumin levels (to define ionized calcium level) and magnesium levels due to retrospective analysis

Therefore, as also highlighted in our paper, the treatment of vitamin D insufficiency and the evaluation of SNHPT before bariatric surgery should be recommended.

The clinical significance of preoperative SNHPT and in particular SNHPT after bariatric surgery remains undefined and further studies are required.

## Figures and Tables

**Table 1:** Inclusion and exclusion criteria for eligibility of the study.

Inclusion criteria	Exclusion criteria
Age between 18 and 60 years	Currently using pharmacological (e.g., hormone replacement therapy) or supplementation treatment to influence VD metabolism
Caucasian ethnicity	Disease influencing calcium metabolism, polyendocrinopathy or autoimmune disease and other causes of secondary hyperparathyroidism^∗^
BMI >40 kg/m^2^ or 35–40 kg/m^2^ with obesity-associated comorbidity	History of cancer
Availability and willingness to comply with 18-month outpatient follow-up	Prior head or neck surgery
Availability and willingness to have blood samples tested for calcium, 25-hydroxy vitamin D, and PTH levels.	Prior abdominal or bariatric surgery
Availability to take oral supplements, if needed	Lack of calcium metabolism documentation
Normal preoperative renal function	Lost to follow-up

^∗^Causes of secondary hyperparathyroidism according to Fraser [22]: (i) steroid therapy; (ii) patients overclothing; (iii) bisphosphonate therapy; (iv) osteolytic lesions; (v) diuretic therapy; (vi) previous acute pancreatitis; (vii) state of sepsis; (viii) chronic kidney disease and hypercalciuria; (ix) intake of drugs that interact with vitamin D (e.g., phenobarbital and phenytoin); and (x) use of antacids containing aluminum that interfere with phosphate absorption.

**Table 2:** Presurgical anthropometric and laboratory variables of study group (*n* = 226).

Variable	Mean ± SD
Age (years)	37 ± 9.09
BMI (kg/m^2^)	44.34 ± 5.06
Elapsed time since last effective diet (years)	9 ± 1.05
25-hydroxy vitamin D (ng/mL)	25.3 ± 4.25
Serum calcium (mg/dL)	9.18 ± 2.01
Serum PTH (pg/dL)	81.48 ± 3.45

**Table 3:** Demographic and laboratory characteristics of presurgical SNHPT patients (*group 1*, *n* = 201) and nonpresurgical SNHPT (*group 2*, *n* = 25) patients.

Presurgical variable	Group 1	Group 2	*p* ^∗^
Age (years, mean ± SD)	31.5 ± 3.5	35.5 ± 2.8	0.123
BMI (kg/m^2^, mean ± SD)	43.4 ± 4.5	39 ± 6.7	0.103
Elapsed time since last effective diet (years, mean ± SD)	8.5 ± 0.5	4 ± 1.2	<0.05
Serum 25-hydroxy vitamin D (ng/mL, mean ± SD)	23.8 ± 2.6	29 ± 1.8	<0.05
Serum calcium (mg/dL, mean ± SD)	9 ± 1.8	9 ± 2.1	0.456
Serum PTH (pg/dL, mean ± SD)	78.8 ± 3.2	63 ± 5.4	<0.05

^∗^ANOVA for continuous variables.

**Table 4:** Anthropometric and laboratory values during follow-up: group 3 patients (*n* = 153) recovered from SNHPT within 6 months after surgery and group 4 patients (*n* = 48) within 18 months; group 2 patients (*n* = 25) without presurgical SNHPT.

	Variable	Group 4	Group 3	*p* ^∗^ (group 4 versus group 3)	Group 2
6 months	Serum 25-hydroxy vitamin D (ng/mL, mean ± SD)	25.5 ± 2.5	35.6 ± 1.8	*N.S*.	37.1 ± 3.1
	Serum calcium (mg/dL, mean ± SD)	9.1 ± 1.0	9.08 ± 1.1	*N.S.*	9.1 ± 1.7
	Serum PTH (ml/dL, mean ± SD)	74.56 ± 5.2	50.7 ± 3.5	<0.05	49.3 ± 2.5
	Elapsed time since last effective diet (years, mean ± SD)	12 ± 1.5	5 ± 0.5	<0.05	4 ± 1.2
	Extra weight-loss percentage (%EWL, mean ± SD)	44.1 ± 2.5	40.3 ± 2.2	<0.05	38.9 ± 1.8

12 months	Serum 25-hydroxy vitamin D (ng/mL, mean ± SD)	26.4 ± 3.2	42.4 ± 1.7	*N.S*.	46.1 ± 1.3
	Serum calcium (mg/dL, mean ± SD)	9.3 ± 0.5	9.08 ± 0.9	*N.S*.	9.1 ± 1.1
	Serum PTH (mg/dL, mean ± SD)	73 ± 3.2	43.1 ± 3.3	<0.05	45.6 ± 1.4
	Extra weight loss percentage (%EWL, mean ± SD)	54.3 ± 1.5	46.6 ± 3.1	<0.05	46.5 ± 1.9

18 months	Serum 25-hydroxy vitamin D (ng/mL, mean ± SD)	32.1 ± 0.9	43.7 ± 1.2	*N.S*.	46.3 ± 1.9
	Serum calcium (mg/dL, mean ± SD)	9.8 ± 1.1	9.6 ± 2.0	*N.S*.	9.4 ± 1.1
	Serum PTH (mg/dL, mean ± SD)	56.6 ± 2.2	40.3 ± 1.5	<0.05	39.1 ± 0.5
	Extra weight loss percentage (%, mean ± SD)	65.3 ± 2.6	59.1 ± 3.2	<0.05	59.6 ± 2.8

^∗^ANOVA for continuous variables; N.S. not statistically significant.

## Additional Points

*Answer the Study Importance Questions*. It is well known that patients could develop nutritional deficiencies after bariatric surgery. Secondary normocalcemic hyperparathyroidism is a clinical condition related to morbid obesity and needs to be stabilized prior to surgery. We analyzed predictive factors of secondary normocalcemic hyperparathyroidism in a large cohort of patients submitted to bariatric surgery. We established the importance of the presurgical diet in the resolution of SNHPT.

## References

[1] Ernst B., Thurnheer M., Schmid S. M., Schultes B. (2009). Evidence for the necessity to systematically assess micronutrient status prior to bariatric surgery. *Obesity Surgery*.

[2] Stein E. M., Strain G., Sinha N. (2009). Vitamin D insufficiency prior to bariatric surgery: risk factors and a pilot treatment study. *Clinical Endocrinology*.

[3] Gallagher J. C., Yalamanchili V., Smith L. M. (2013). The effect of vitamin D supplementation on serum 25OHD in thin and obese women. *The Journal of Steroid Biochemistry and Molecular Biology*.

[4] Karefylakis C., Näslund I., Edholm D., Sundbom M., Karlsson F. A., Rask E. (2014). Vitamin D status 10 years after primary gastric bypass: gravely high prevalence of hypovitaminosis D and raised PTH levels. *Obesity Surgery*.

[5] Ybarra J., Sánchez-Hernández J., Gich I. (2005). Unchanged hypovitaminosis D and secondary hyperparathyroidism in morbid obesity after bariatric surgery. *Obesity Surgery*.

[6] Valderas J. P., Velasco S., Solari S. (2009). Increase of bone resorption and the parathyroid hormone in postmenopausal women in the long-term after Roux-en-Y gastric bypass. *Obesity Surgery*.

[7] Fish E., Beverstein G., Olson D., Reinhardt S., Garren M., Gould J. (2010). Vitamin D status of morbidly obese bariatric surgery patients. *Journal of Surgical Research*.

[8] Ducloux R., Nobécourt E., Chevallier J. M., Ducloux H., Elian N., Altman J. J. (2011). Vitamin D deficiency before bariatric surgery: should supplement intake be routinely prescribed?. *Obesity Surgery*.

[9] Feldman D., Pike J. W., Glorieux F. (2005). *Vitamin D*.

[10] Coupaye M., Breuil M. C., Rivière P. (2013). Serum vitamin D increases with weight loss in obese subjects 6 months after Roux-en-Y gastric bypass. *Obesity Surgery*.

[11] Youssef Y., Richards W. O., Sekhar N. (2007). Risk of secondary hyperparathyroidism after laparoscopic gastric bypass surgery in obese women. *Surgical Endoscopy*.

[12] Harris S. S. (2006). Vitamin D and African Americans. *The Journal of Nutrition*.

[13] Clements R. H., Yellumahanthi K., Wesley M., Ballem N., Bland K. I. (2008). Hyperparathyroidism and vitamin D deficiency after laparoscopic gastric bypasS. *The American Surgeon*.

[14] Flores L., Martínez Osaba M. J., Andreu A., Moizé V., Rodríguez L., Vidal J. (2010). Calcium and vitamin D supplementation after gastric bypass should be individualized to improve or avoid hyperparathyroidism. *Obesity Surgery*.

[15] Gasteyger C., Suter M., Gaillard R. C., Giusti V. (2008). Nutritional deficiencies after roux-en-Y gastric bypass for morbid obesity often cannot be prevented by standard multivitamin supplementation. *The American Journal of Clinical Nutrition*.

[16] Hage M. P., el-Hajj Fuleihan G. (2014). Bone and mineral metabolism in patients undergoing Roux-en-Y gastric bypass. *Osteoporosis International*.

[17] Grethen E., Hill K. M., Jones R. (2012). Serum leptin, parathyroid hormone, 1,25-dihydroxyvitamin D, fibroblast growth factor 23, bone alkaline phosphatase, and sclerostin relationships in obesity. *The Journal of Clinical Endocrinology & Metabolism*.

[18] Tsiftsis D. D. A., Mylonas P., Mead N., Kalfarentzos F., Alexandrides T. K. (2009). Bone mass decreases in morbidly obese women after long limb-biliopancreatic diversion and marked weight loss without secondary hyperparathyroidism. A physiological adaptation to weight loss?. *Obesity Surgery*.

[19] Balsa J. A., Botella-Carretero J. I., Peromingo R. (2010). Chronic increase of bone turnover markers after biliopancreatic diversion is related to secondary hyperparathyroidism and weight loss. Relation with bone mineral density. *Obesity Surgery*.

[20] Rosen C. J. (2011). Vitamin D insufficiency. *The New England Journal of Medicine*.

[21] Pitroda A. P., Harris S. S., Dawson-Hughes B. (2009). The association of adiposity with parathyroid hormone in healthy older adults. *Endocrine*.

[22] Fraser W. D. (2009). Hyperparathyroidism. *The Lancet*.

[23] Moreiro J., Ruiz O., Perez G. (2007). Parathyroid hormone and bone marker levels in patients with morbid obesity before and after biliopancreatic diversion. *Obesity Surgery*.

[24] Hamoui N., Anthone G., Crookes P. F. (2004). Calcium metabolism in the morbidly obese. *Obesity Surgery*.

[25] Compston J. E., Vedi S., Ledger J. E., Webb A., Gazet J. C., Pilkington T. R. (1981). Vitamin D status and bone histomorphometry in gross obesity. *The American Journal of Clinical Nutrition*.

[26] Costa T. L., Paganotto M., Radominski R. B., Kulak C. M., Borba V. C. (2015). Calcium metabolism, vitamin D and bone mineral density after bariatric surgery. *Osteoporosis International*.

[27] Jakobsen J., Maribo H., Bysted A., Sommer H. M., Hels O. (2007). 25-Hydroxyvitamin D_3_ affects vitamin D status similar to vitamin D_3_ in pigs – but the meat produced has a lower content of vitamin D. *British Journal of Nutrition*.

[28] Aasheim E. T., Bjorkman S., Sovik T. T. (2009). Vitamin status after bariatric surgery: a randomized study of gastric bypass and duodenal switch. *The American Journal of Clinical Nutrition*.

[29] Signori C., Zalesin K. C., Franklin B., Miller W. L., McCullough P. A. (2010). Effect of gastric bypass on vitamin D and secondary hyperparathyroidism. *Obesity Surgery*.

[30] De Prisco C., Levine S. N. (2005). Metabolic bone disease after gastric bypass surgery for obesity. *The American Journal of the Medical Sciences*.

[31] Johnson J. M., Maher J. W., DeMaria E. J., Downs R. W., Wolfe L. G., Kellum J. M. (2006). The long-term effects of gastric bypass on vitamin D metabolism. *Annals of Surgery*.

[32] Rydén A., Torgerson J. S. (2006). The Swedish Obese Subjects Study—what has been accomplished to date?. *Surgery for Obesity and Related Diseases*.

[33] Cariani S., Vittimberga G., Grani S., Lucchi A., Guerra M., Amenta E. (2003). A functional Roux-en-Y gastric bypass to avoid gastric exclusion: 1-year results. *Obesity Surgery*.

[34] Karlsson J., Taft C., Rydén A., Sjöström L., Sullivan M. (2007). Ten-year trends in health-related quality of life after surgical and conventional treatment for severe obesity: the SOS intervention study. *International Journal of Obesity*.

[35] Sicob (2008). *Italian Guidelines*.

[36] Sicob (2016). *Italian Guidelines*.

[37] Grethen E., McClintock R., Gupta C. E. (2011). Vitamin D and hyperparathyroidism in obesity. *The Journal of Clinical Endocrinology & Metabolism*.

[38] Sánchez-Hernández J., Ybarra J., Gich I. (2005). Effects of bariatric surgery on vitamin D status and secondary hyperparathyroidism: a prospective study. *Obesity Surgery*.

[39] Goode L. R., Brolin R. E., Chowdhury H. A., Shapses S. A. (2004). Bone and gastric bypass surgery: effects of dietary calcium and vitamin D. *Obesity Research*.

[40] Mechanick J. I., Youdim A., Jones D. B. (2013). Clinical practice guidelines for the perioperative nutritional, metabolic, and nonsurgical support of the bariatric surgery patient—2013 update: cosponsored by American Association of Clinical Endocrinologists, The Obesity Society, and American Society for Metabolic & Bariatric Surgery. *Obesity*.

[41] Levinson R., Silverman J. B., Catella J. G., Rybak I., Jolin H., Isom K. (2013). Pharmacotherapy prevention and management of nutritional deficiencies post Roux-en-Y gastric bypass. *Obesity Surgery*.

